# Supplementary material for: Tunable Tensile Properties of Polypropylene and Polyethylene Terephthalate Fibrillar Blends through Micro-/Nanolayered Extrusion Technology
Source: Polymers (Basel). 2020 Nov 4;12(11):2585. doi: 10.3390/polym12112585 (PMC7694209; doi:10.3390/polym12112585)
Supplement: Supplementary file 1 [file polymers-12-02585-s001.pdf]

polymers

MDPI

Supplementary Material

Tunable Tensile Properties of Polypropylene and Polyethylene Terephthalate Fibrillar Blends Through Micro-/Nanolayered Extrusion Technology

Mahmoud Embabi, Mu Sung Kweon, Zuolong Chen and Patrick C. Lee *

Multifunctional Composites Manufacturing Laboratory (MCML), Department of Mechanical and Industrial Engineering, University of Toronto, 5 King's College Road, Toronto, ON M5S 3G8, Canada; embabi@mie.utoronto.ca (M.E.); mkweon@mie.utoronto.ca (M.S.K.); zchen@mie.utoronto.ca (Z.C.)

* Correspondence: patricklee@mie.utoronto.ca

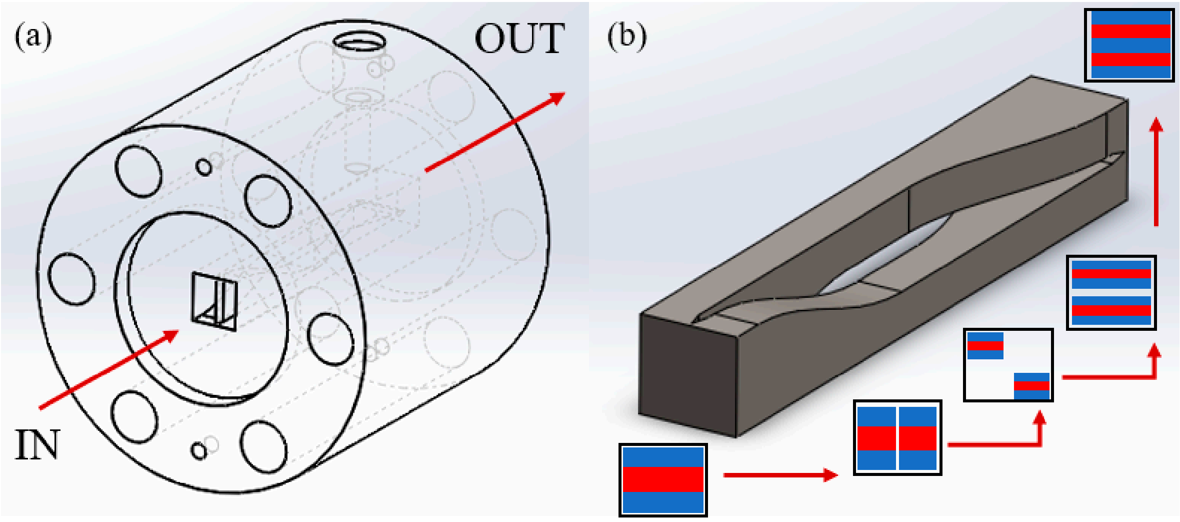

Figure S1. (a) Schematic representation of a single layer multiplier and (b) layer multiplication mechanism with an A-B-A inlet layer setting.

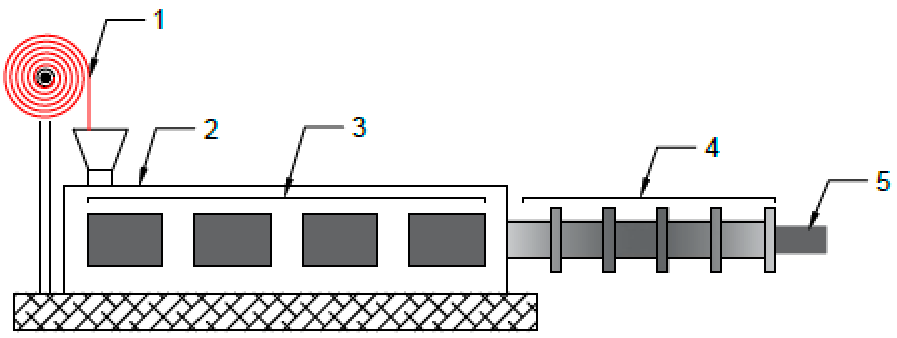

Figure S2. Schematic representation of the MNL extrusion system: (1) nonwoven fiber mat, (2) twin-screw extruder, (3) heating zones, (4) layer multipliers, (5) extrudate.

Differential scanning calorimetry (DSC)

DSC was performed on PP/PET fibrillar blends of 3, 7, and 15 wt.% PET concentrations to investigate the appropriate MNL extrusion processing window (Figure S3). Samples were prepared by cutting a PP/PET fiber mat into a 5–10 mg disk placed in an aluminum pan. Prior to applying the heating ramp, each sample was equilibrated to 40 °C. A ramp rate of 10 °C/min was used to heat the samples to 300 °C to determine the end melting temperature of the PP matrix (~175°C) and the onset melting temperature of the PET nanofibers (~240 °C). The first heating curve was used for analysis as this is representative of the thermogram of the non-woven fiber mat roll to be processed in MNL extrusion. Measurements were repeated twice for each PET concentration by preparing samples from different sections of the fiber mat. Identical heating curves were obtained at each PET concentration, indicating that the measurements were reproducible.

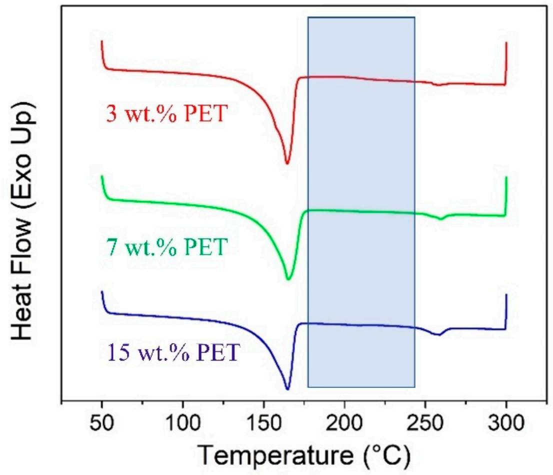

Figure S3. First heating thermograms of PP/PET fibrillar blends with 3, 7, and 15 wt.% PET contents. Shaded region indicates the MNL extrusion processing window.

Shear Rheology

The melt rheology of the PP matrix was obtained from small amplitude oscillatory shear (SAOS) measurements in an ARES-G2 rheometer (TA Instruments). PP pellets were compressed molded at 200 °C for 5 min into a disk with a thickness of 1 mm. A parallel plate geometry was employed, and frequency sweep measurements were conducted at 200 °C from 0.1 to 628 rad/s under a 10% strain, which was verified to be well within the linear viscoelastic regime as determined from strain sweep experiments. The Cox–Merz rule was applied to the SAOS data to estimate the shear viscosity as a function of shear rate (Figure S4).

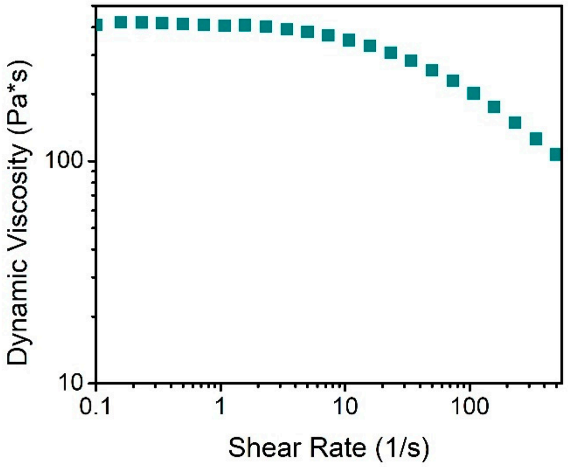

Figure S4. Dynamic viscosity of PP at 200.

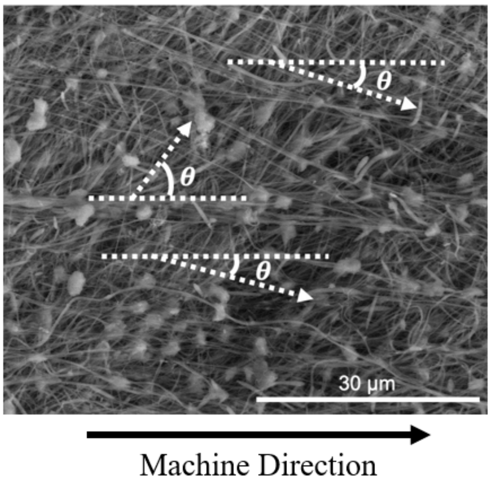

Figure S5. An SEM image showing the exposed PET fibers after xylene etching and the angle of nanofiber orientation (θ) with respect to the MD (horizontal as shown).
